# Geometric constraints and semantic optimization SLAM algorithm for dynamic scenarios

**DOI:** 10.1038/s41598-025-16714-x

**Published:** 2025-08-29

**Authors:** Yanli Liu, Yuting Wang, Heng Zhang, Qi Li

**Affiliations:** School of Electronic Information, Shanghai DianJi University, Shanghai, 201306 China

**Keywords:** Visual SLAM, Dynamic feature filtering, Semantic information, Geometric constraints, Computer science, Information technology

## Abstract

Traditional visual SLAM systems are predominantly designed for static environments, where they encounter challenges in dynamic scenes, leading to increased system errors and redundancy. This paper introduces a dynamic feature detection and filtering algorithm. Through a feature point selection and optimization strategy within quadtree nodes, high-response feature points are prioritized. Semantic information is leveraged to remove features on prior dynamic objects, and geometric constraints are applied to filter truly dynamic features. For unmatched features, an extension method is used, and high-confidence points are weighted to obtain feature point status information. Compared with the standard ORB-SLAM2 algorithm, our improved algorithm achieves over a 90% performance increase in highly dynamic environments, with absolute trajectory error performance improvements up to 96.84% in low-dynamic settings. Overall, our algorithm demonstrates superior adaptability and robustness in dynamic environments.

## Introduction

With the rapid advancement of intelligent robotics, mobile robots have been increasingly deployed in various fields, including autonomous driving, drone navigation, indoor service robotics, and search and rescue operations. In these complex and dynamic environments, precise localization and environmental perception are critical for autonomous navigation. Simultaneous Localization and Mapping (SLAM) has emerged as a fundamental technology in this domain, attracting significant research interest.

SLAM aims to construct a spatial map while simultaneously estimating the robot’s position within it. This process relies on sensors such as cameras, LiDAR, and depth sensors, enabling robots to perform real-time mapping and localization. By leveraging SLAM, robots can autonomously plan paths, avoid obstacles, and execute tasks in unknown environments. The technology has demonstrated considerable potential in applications such as autonomous vehicle navigation, drone-based environmental exploration, precise positioning of indoor service robots, and complex rescue missions.

Despite the advancements in SLAM, most traditional visual SLAM systems (e.g., Direct Sparse Odometry ^1^, Large-Scale Direct Monocular SLAM ^2^, and ORB-SLAM2 ^3^) operate under the assumption of a static environment where objects remain stationary. However, real-world environments are inherently dynamic, with moving pedestrians and vehicles on streets, human activity and pets indoors, and animals in natural settings. These dynamic elements pose significant challenges to SLAM systems.

The presence of moving objects can cause instability in visual feature points, leading to erroneous feature matching between frames and accumulated pose estimation errors ^4^. Furthermore, if dynamic features are incorrectly classified as part of a static scene, the generated map may significantly deviate from reality, resulting in distortions that affect path planning and navigation accuracy. In highly dynamic environments, such as crowded shopping malls or busy urban streets, these challenges can further degrade the robustness of SLAM systems and, in extreme cases, cause system failure.

This paper addresses the challenges of localization accuracy and robustness faced by SLAM systems in complex dynamic environments. An innovative feature detection and filtering algorithm is proposed to significantly enhance the performance of SLAM systems in dynamic settings. Traditional visual SLAM systems are vulnerable to interference from moving objects in dynamic environments, leading to feature point matching errors, pose estimation inaccuracies, and map distortion. This study combines semantic information and geometric constraints ^5^, introducing a new dynamic feature filtering technique aimed at solving problems such as uneven feature extraction, misidentification of dynamic features, and low feature point matching rates in dynamic scenes.

To address the issue of uneven feature extraction, this method optimizes the distribution of Qtree_ORB feature points by limiting the depth of quadtree node division, thus improving the stability of feature extraction. In the feature filtering phase, this study prioritizes retaining feature points with high response values to ensure the SLAM system can utilize higher-quality feature information for pose estimation. Additionally, semantic information is introduced to accurately identify known dynamic objects (such as pedestrians and vehicles), removing feature points from these dynamic objects to avoid interference with the accuracy of static scene mapping. Furthermore, the application of geometric constraints enhances the accuracy of dynamic feature filtering by detecting real dynamic feature points based on geometric properties, thus reducing the risk of mismatches.

For unmatched feature points during the matching process, this paper proposes a feature point extension-based processing method. By performing weighted calculations within an expanded region with high confidence, this method effectively retrieves the state information of unmatched feature points, improving the feature matching success rate and enhancing the SLAM system’s real-time performance and robustness in dynamic environments. Experiments conducted on the TUM dataset ^6^ validate the effectiveness of the improved algorithm. The results show that the proposed method significantly optimizes the localization accuracy and map construction quality of SLAM systems in complex dynamic scenes, demonstrating great potential for practical applications.

The main contributions of this study can be summarized as follows:

1. An improved Qtree_ORB feature extraction algorithm is introduced to ORB-SLAM2. This method adopts feature selection and optimization strategies in quadtree nodes, addressing the limitations of existing methods, alleviating uniformity issues in feature extraction, and improving the computational efficiency of the algorithm.
2. The combination of semantic information and geometric constraints enables precise filtering of dynamic feature points, enhancing the accuracy, stability, and robustness of the visual SLAM system while improving real-time performance.
3. A cluster-based feature point mismatch handling strategy is proposed for the feature matching algorithm in ORB-SLAM2. For unmatched points, the method processes them by expanding the region and obtaining their state information via high-confidence weighted calculations.

## Related work

### Research on feature point extraction of visual SLAM

The motion of dynamic objects interferes with feature point matching, causing localization errors. In dynamic scenes, the system struggles to distinguish between static backgrounds and dynamic objects. Feature point extraction is crucial in visual SLAM, and the choice of extraction method directly affects system performance. Traditional visual SLAM algorithms mainly rely on point features, such as SIFT ^7^, ORB ^8^, SURF ^9^, and other methods. To enhance system robustness and accuracy, recent studies have begun incorporating line features, such as the integration of point, line, and surface features in the PLP-SLAM algorithm ^10^.

Smith et al. propose the SUSAN ^11^ algorithm, which determines corner points by comparing pixel values in the neighborhood of the template center. This method avoids gradient operations, offering strong noise resistance but is sensitive to threshold settings. In contrast, ORB features excel in efficient extraction and matching, outperforming SIFE and SURF in real-time performance, though the extracted feature points are unevenly distributed.

The standard ORB algorithm is popular for its real-time performance but suffers from “clustering” in feature extraction, with most points concentrated in high-texture areas and few in low-texture areas. This leads to incomplete representation of the image, affecting pose estimation and trajectory tracking in SLAM. To address the uneven distribution of ORB feature points, Raúl et al. ^3^^,^ ^12^ introduce a quadtree structure and adaptive thresholds, improving uniformity. However, this approach requires multiple iterations for optimal results and can lead to over-uniformity, reducing feature density and affecting performance. Bei Q et al. ^13^ propose an improved algorithm with adaptive thresholds to enhance feature extraction in low-texture regions but did not address the uniformity issue, leaving the uneven distribution unresolved.

To address the limitations of the Qtree_ORB algorithm, this paper proposes a method that limits the depth of quadtree nodes, alleviating the issue of overly uniform feature distribution. This improves the balance of feature distribution while significantly enhancing the algorithm’s efficiency.

### Research on dynamic point filtering of visual SLAM

Visual SLAM faces challenges in dynamic environments, where moving objects reduce pose estimation accuracy and map quality. Improving localization accuracy and minimizing the impact of dynamic objects have become key research areas, with solutions mainly categorized into the following types.

1. Filtering method based on motion model Fang Y et al. ^14^ propose a technique combining point matching and uniform sampling to calculate optical flow by analyzing pixel motion between frames. Dynamic feature points are identified and filtered when their flow deviates from the overall scene. Mohamed et al. ^15^ introduce an improved optical flow method using scene flow and region-growing algorithms to separate dynamic and static objects, enhancing feature matching accuracy. However, its high computational complexity limits real-time performance in high-frequency dynamic scenes.
2. Filtering method based on geometric constraints Wang et al. ^16^ combine mathematical models with geometric constraints to detect dynamic outliers in scenes. By applying the K-means algorithm to depth image data, outliers were clustered, and regions with outliers exceeding a set threshold were identified and segmented as dynamic objects.
3. Filtering method based on multi-sensor fusion Zou D et al. ^17^ propose Co-SLAM, which shares feature points across multiple cameras and integrates inter-camera pose estimation and mapping to handle dynamic objects. Its accuracy depends on camera count: fewer cameras reduce segmentation performance, while too many increase computational load. Kim et al. ^18^ combine RGB-D cameras with IMU data, fusing depth and RGB information to compute motion vectors for 3D feature points, classifying them as static or dynamic. IMU integration improved pose estimation accuracy, effectively reducing the impact of dynamic objects on the SLAM system.
4. Filtering method based on deep learning Bescos et al. ^19^ propose DynSLAM, using Mask R-CNN for dynamic object detection and static map reconstruction. Vincent et al. ^20^ enhance pose estimation and mapping with Detectron2 and Kalman filtering. RDMO-SLAM ^21^ improve real-time performance by applying semantic segmentation to keyframes and incorporating optical flow.

## Method

### Improved Qtree_ORB feature point equalization strategy

The ORB algorithm enhances feature extraction by selecting the top N FAST feature points with the highest Harris ^22^ responses. This approach ensures computational efficiency while preserving high-quality feature points. However, in texture-rich and dense scenes, feature points tend to cluster locally, limiting their ability to capture global information. To improve the Qtree_ORB feature extraction in ORB-SLAM2 ^3^, we propose a method that restricts the depth of quadtree node division. This adjustment helps balance feature distribution and prevents excessive uniformity.

#### Standard Qtree_ORB feature point algorithm

The standard ORB algorithm improves FAST ^23^ feature points and BRIEF ^24^ descriptors by incorporating directional information, ensuring rotational invariance. ORB features are widely used in SLAM systems and consist of the following components:

1. Image Partitioning and Feature Detection: The input image is initially divided into large sub-blocks using a quadtree method, recursively splitting it into four equal regions. This ensures a more uniform distribution of feature points. FAST (Features from Accelerated Segment Test) is then applied within each sub-block to detect potential feature points by analyzing brightness variations around pixels.
2. Harris Response Calculation: The Harris response value is computed for each detected FAST feature point to assess corner strength. This helps identify stable and significant feature points for selection.
3. Feature Selection and Orientation Assignment: The top N feature points with the highest Harris responses are selected within each sub-block, ensuring a more even distribution. A primary orientation is then assigned based on the gradient direction, enhancing rotational invariance.
4. BRIEF Descriptor Calculation: The BRIEF (Binary Robust Independent Elemental Features) descriptor is computed for each feature point. By comparing pixel pairs around the point, BRIEF provides high computational efficiency and requires minimal storage.
5. Quadtree Splitting: If a sub-block contains more feature points than a predefined threshold, it is further divided. This process continues until each sub-block meets the required criteria, preventing excessive clustering in texture-rich areas. Finally, feature points from all sub-blocks are merged to achieve a balanced distribution across the image.

**Fig. 1:**
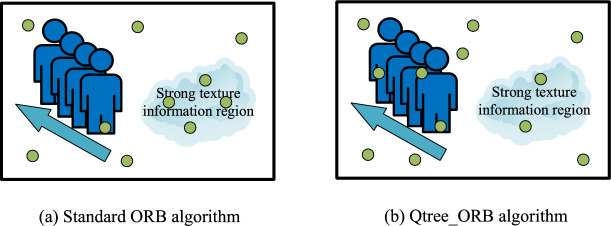
ORB feature extraction in complex scenarios.

As shown in the following Fig. 1, the standard ORB algorithm has limitations in complex scenes, particularly those with fast-moving vehicles, pedestrians, and strong texture regions. Motion blur caused by rapid movement reduces grayscale differences between pixels, lowering the quality of extracted features and affecting tracking performance. Additionally, standard ORB tends to concentrate feature points in texture-rich regions, limiting its ability to capture global information ^25^. This imbalance can introduce errors in SLAM systems. Therefore, improving the uniform distribution of ORB feature points is crucial for enhancing the accuracy and robustness of SLAM.

#### Improved Qtree_ORB feature point algorithm

To overcome the limitations of the standard Qtree_ORB feature point algorithm, we propose an improved strategy. A multi-resolution image pyramid is constructed to capture multi-scale information, effectively addressing scale variations in images. Additionally, the image pyramid optimally distributes feature points across its layers, enhancing their spatial distribution. Specifically, the number of feature points extracted at each layer is set as follows:

1 $$\begin{aligned} \operatorname {DesF}_{i}=N \times \text {DesF}_i = N \times \frac{1 - \text {InvSF}}{1 - \text {InvSF}^n} \times \text {InvSF}^i \end{aligned}$$

Where *N* represents the total number of anticipated feature points, *n* is the total number of pyramid layers, $\operatorname {DesF}_{i}$ represents the total number of features to be extracted from the I-layer pyramid, and $\operatorname {InvSF}$ represents the reciprocal of the scale element. This configuration allows for a balanced allocation of feature points across each layer, ensuring a more uniform distribution among feature points at different scales.

In ORB-SLAM2, the fixed threshold for FAST feature extraction cannot effectively adapt to changes in the external environment. The improved algorithm calculates an adaptive threshold based on image grayscale values, dynamically adjusting the feature extraction criteria according to lighting variations and image characteristics. This ensures stable and accurate feature point extraction even in scenes with uneven illumination or significant texture changes. The adaptive threshold enhances robustness, enabling meaningful feature points to be extracted under different lighting conditions, especially in highly dynamic environments. To achieve this, the extraction threshold is adaptively adjusted, with the initial threshold set as follows:

2 $$\begin{aligned} \text { iniTh }=\frac{1}{k \cdot n i} \sum _{x=1}^{n i}(I(x)-k)^{2} \end{aligned}$$

In the method, $\text{ iniTh }$ acts for the gray value of the x-th pixel, *k* is the standard gray value of all pixels in the image and *ni* is the total number of pixels. Unlike the standard algorithm, the improved method restricts the depth of quadtree node division when partitioning the image. By limiting the quadtree node splitting depth, the algorithm ensures a uniform distribution of feature points without making them too sparse. This improves the comprehensiveness and effectiveness of feature extraction. The relationship between the number of nodes in the quadtree and the depth of the division can be expressed as follows:

3 $$\begin{aligned} N=4^{d} \end{aligned}$$

Where *N* is the number of quadtree nodes and *d* represents the partition depth.

The core of the improved algorithm lies in the feature retention strategy within quadtree nodes. If a node contains only one feature point, it is directly retained. For nodes with two or more feature points, the Harris response values are first sorted, and the point with the highest response is kept while the retained point is removed from the node. This process is repeated until the number of retained feature points meets the expected requirement.

Additionally, the improved algorithm imposes a minimum threshold on the Harris response value of feature points. If a feature point’s Harris response falls below this threshold, no further feature points will be extracted from that node in subsequent iterations. The Harris response value measures the stability and significance of a feature point, with higher values indicating greater stability and a stronger representation of key image regions. By introducing a minimum threshold, the algorithm effectively eliminates low-quality and unreliable feature points, improving extraction accuracy and reducing mismatches and localization errors caused by weak feature points.

Algorithm 1 provides a detailed description of the node division and feature retention strategies.

**Algorithm 1:**
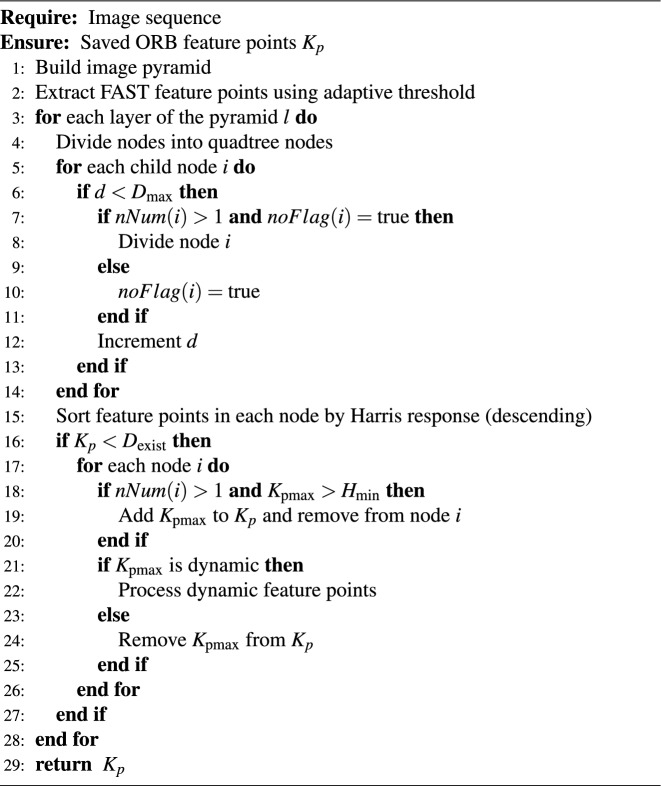
Improved Qtree_ORB Feature Point Homogenization Algorithm (Simplified)

This improvement prioritizes feature points with higher Harris response values within the nodes, effectively filtering out dynamic feature points and enhancing the integrity of the feature points and the stability of the SLAM system.

### Dynamic feature point filtering algorithm combining geometric and semantic constraints

**Fig. 2:**
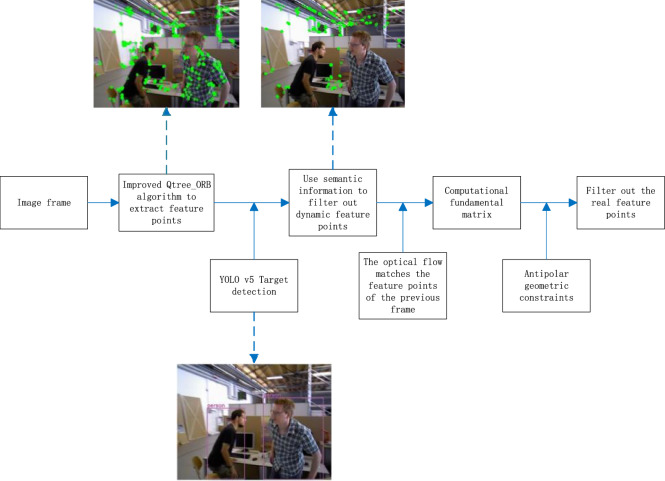
Dynamic feature point filtering process of the SGC algorithm.

The semantic and geometric constraints dynamic features filter algorithm (SGC) is an improved method for dynamic feature detection. It extracts and optimizes feature points using the improved Qtree_ORB algorithm, leverages YOLOv5 to detect prior dynamic targets, filters dynamic feature points, and applies geometric constraints to eliminate true dynamic points, ensuring stable and accurate feature extraction. Figure 2 is the dynamic feature point filtering process of the SGC algorithm.

#### Semantic information extraction based on object detection network YOLOv5

In SLAM systems, semantic information refers to high-level data related to object categories, attributes, or relationships in the environment, extending beyond spatial geometric features. This semantic understanding enhances environmental perception for robots or autonomous vehicles, enabling more accurate localization and navigation in complex scenarios. With the rapid development of deep learning, object detection has become widely adopted for various tasks. Its primary function involves identifying target objects in images and marking their precise locations, providing fundamental support for subsequent analysis. This study employs YOLOv5 as the semantic information extraction network. YOLOv5 demonstrates powerful and efficient detection capabilities, offering faster training, higher inference efficiency, and suitability for resource-constrained devices, making it ideal for real-time detection in complex environments ^26^.

Building upon ORB-SLAM2, we introduce an additional semantic detection thread using YOLOv5 to identify prior dynamic objects in input frames. The detection results are transmitted to the tracking thread to eliminate feature points within dynamic regions during preprocessing, thereby improving camera pose estimation accuracy. In indoor environments, humans and animals are typically considered primary dynamic targets, where their associated feature points are classified as dynamic. Removing these points through YOLOv5 detection enhances SLAM system precision. However, this approach introduces challenges. YOLOv5 may exhibit detection failures for small objects and partially occluded targets ^27^, potentially affecting SLAM’s environmental perception, particularly in applications requiring accurate distant obstacle recognition. Detection bounding boxes might also encompass static objects like chairs or monitors. Moreover, dynamic targets in indoor environments are not limited to humans. For instance, rotating chairs manipulated by users could also become dynamic objects, indicating the incompleteness of considering only humans as dynamic targets.

#### Semantic information constraints and geometric constraints of dynamic feature points

This paper proposes an improved dynamic point removal algorithm that integrates semantic information and geometric constraints.

First, the improved Qtree_ORB algorithm is used to extract feature points from image frames. By limiting the depth of the quadtree, the algorithm ensures a more balanced distribution of feature points across the image, making it easier to distinguish between dynamic and static points.

Next, the extracted ORB feature points are filtered using semantic information generated by YOLOv5 to remove prior dynamic points. A semantic detection thread is integrated into ORB-SLAM2 to analyze the input image frames, where ORB-SLAM2 filters out prior dynamic feature points based on the object regions identified by YOLOv5. This study focuses on indoor environments, where humans and animals are predefined as dynamic objects. The semantic thread passes the detected dynamic object information to the tracking thread as prior knowledge. Once feature point extraction is complete, dynamic feature points are removed based on this prior information.

Then, optical flow is employed to track the remaining feature points. Optical flow is well-suited for capturing dynamic changes in images, as it estimates pixel motion to interpret object movement. Since it only tracks FAST keypoints without requiring descriptor matching, it offers good real-time performance. Therefore, optical flow is applied to track the remaining feature points efficiently. In this study, the Lucas-Kanade (LK) optical flow method is employed for feature point tracking. The core idea of this method is to estimate optical flow within small local regions of the image, assuming that the motion within each local window is consistent. The LK optical flow method is based on the assumption that the brightness of each point in the image remains constant over time during object motion, expressed as:

4 $$\begin{aligned} I(x,y,t)=I(x+\triangle x, y+\triangle y, t+\triangle t) \end{aligned}$$

Where *I*(*x*, *y*, *t*) represents the intensity of the point (*x*, *y*)) in the image at time *t*, $\triangle x$ and $\triangle y$ represent the displacement of the point within the time interval $\triangle t$. Based on the brightness constancy assumption, a Taylor series expansion is applied to the image over time, yielding:

5 $$\begin{aligned} I_{x}u+I_{y}v+I_{t}=0 \end{aligned}$$

In the equation, $I_{x}$ represents the image gradient in the x-direction, indicating the rate of change in image intensity along the horizontal axis. $I_{y}$ denotes the image gradient in the y-direction, representing the rate of change in intensity along the vertical axis. $I_{t}$ is the temporal gradient of the image, describing the variation of intensity over time. *u* and *v* correspond to the optical flow components (pixel motion velocity) in the horizontal and vertical directions, respectively. Within each local region, the optical flow equation is applied to all pixels in the window, and a least-squares optimization is performed to estimate the optical flow for each point, given by:

6 $$\begin{aligned} \left[ \begin{array}{ll}I_{x}&I_{y}\end{array}\right] \left[ \begin{array}{c}u \\ v\end{array}\right] =-I_{t} \end{aligned}$$

There are multiple optical flow equations within the window, and the following system of equations is solved:

7 $$\begin{aligned} \sum \begin{bmatrix} I_{x}^{2} & I_{x}I_{y}\\ I_{x}I_{y} & I_{y}^{2} \end{bmatrix}\begin{bmatrix} u \\ v \end{bmatrix}=-\sum \begin{bmatrix} I_{x}\\ I_{y} \end{bmatrix}I_{t} \end{aligned}$$

By solving the equations, the pixel motion velocities *u* and *v* can be obtained, allowing the estimation of a pixel’s position across multiple frames. Although optical flow-based matching effectively reduces mismatches, feature point tracking loss may still occur.

To address this issue, this paper proposes a clustering-based feature point loss matching strategy. Relying solely on semantic information for dynamic point removal is insufficient, as the prior dynamic objects detected by YOLOv5 are predefined and do not necessarily reflect their actual motion state. Therefore, it is necessary to determine the motion state of the detected objects. This is achieved by integrating semantic information with geometric constraints to filter out dynamic feature points.

After tracking and matching feature points using the optical flow method, the spatial relationship between two consecutive frames can be determined. The fundamental matrix *F* represents the relative transformation between these frames and describes the projection changes of spatial points ^28^. However, in dynamic environments, geometric constraints no longer apply to moving points. This means that the pixel coordinates of dynamic points deviate from the constraints imposed by the fundamental matrix, leading to a discrepancy between the pixel points and their corresponding epipolar lines. By measuring this deviation, the motion state of spatial points can be determined, allowing for the identification of dynamic feature points ^29^.

In the constraint of polar geometry, the basic matrix *F* is mainly calculated. *F* is the algebraic expression of polar geometry. Generally, *F* is:

8 $$\begin{aligned} F=\left( \begin{array}{lll}f_{11} & f_{12} & f_{13} \\ f_{21} & f_{22} & f_{23} \\ f_{31} & f_{32} & f_{33}\end{array}\right) \end{aligned}$$

The basic matrix is generally satisfied $p_{2}^{T}Fp_{1}=0$. From the antipolar geometric constraints:

9 $$\begin{aligned} x_{1}^{T} F x_{2}=\left[ \begin{array}{lll}u_{1}&v_{1}&1\end{array}\right] \left[ \begin{array}{lll}f_{11} & f_{12} & f_{13} \\ f_{21} & f_{22} & f_{23} \\ f_{31} & f_{32} & f_{33}\end{array}\right] \left[ \begin{array}{c}u_{2} \\ v_{2} \\ 1\end{array}\right] =0 \end{aligned}$$

Then corresponding equation:

10 $$\begin{aligned} u_1 u_2 f_{11} + u_1 u_2 f_{12} + u_1 f_{13} + v_1 u_2 f_{21} + v_1 v_2 f_{22} + v_1 f_{23} + u_2 f_{31} + v_2 f_{32} + f_{33} = 0 \end{aligned}$$

In the above formulation, the fundamental matrix *F* has nine unknowns but only eight degrees of freedom. Therefore, it can be estimated using eight pairs of feature points. Analysis shows that the accuracy of *F* depends on the quality of these eight sample points. Thus, preprocessing of feature points is necessary before estimation.

In this section, semantic information of feature points is used for preprocessing. First, feature points associated with prior dynamic objects are removed from the sample set. Then, the RANSAC algorithm is applied to estimate the fundamental matrix *F*. Once an accurate *F* is obtained, the epipolar constraint is imposed on all feature points. By calculating the distance between feature points and their corresponding epipolar lines, truly dynamic feature points can be further filtered out, improving system accuracy and robustness.

### Cluster-enhanced robust feature point matching strategy

To address feature point mismatches between consecutive frames, this paper proposes a cluster-based loss matching strategy, optimizing the tracking process to improve matching stability and accuracy. ORB extracts feature points, followed by initial matching using optical flow. Unmatched points are clustered to expand the matching range. Two dynamic point probability calculations are performed: the first marks dynamic points, and the second filters highly dynamic points. This approach improves feature point matching accuracy, enhancing SLAM system robustness and precision in dynamic environments. By calculating the dynamic probability of each feature point, the algorithm effectively distinguishes between static and dynamic object feature points. Setting an appropriate dynamic probability threshold helps the system eliminate feature points from dynamic objects, preventing mismatches caused by these points. The detailed process is shown in algorithm 2.

**Algorithm 2:**
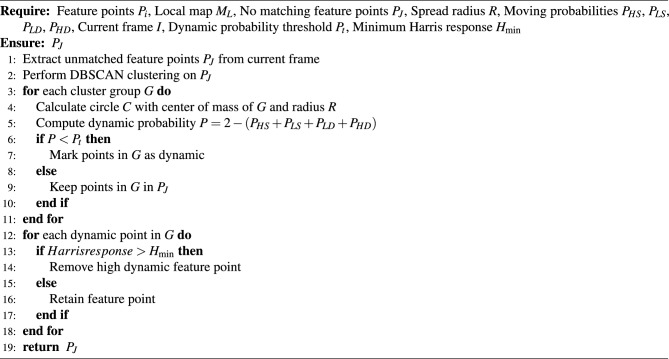
Clustering Feature Point Matching (Simplified)

## Experiments

In this section, we use the TUM dataset ^6^ to test the accuracy and performance of our algorithm. At the same time, we also conducted ablation experiments to further verify its efficiency.

### Experimental environment

The experiment uses a machine cart with an Intel D435i depth camera and lidar. The test environment includes Ubuntu 20.04 and an Nvidia GeForce RTX 3090 GPU. Experiments on the TUM dataset target scenes with varying dynamics, using walking and sitting sequences for high- and low-dynamic environments. Tests were conducted across camera movement patterns: translation (XYZ), rotation (rpy), static (static), and semi-dynamic (half).

### Experimental results

In the dynamic feature point removal experiments for dynamic visual SLAM, Absolute Trajectory Error (ATE) and Relative Pose Error (RPE) are adopted as the primary evaluation metrics. ATE measures the global deviation between the estimated trajectory and the ground truth, providing a comprehensive assessment of the overall accuracy and cumulative drift. In contrast, RPE evaluates the relative motion between consecutive frames, which is particularly effective in capturing short-term tracking stability and local drift behavior. The combined use of ATE and RPE enables a complementary evaluation from both global and local perspectives, thereby avoiding the potential bias of a single-metric assessment.

To quantify these metrics, Root Mean Square Error (RMSE), Standard Deviation (SD), and Mean are employed as statistical indicators. Table 1 details the three parameters:

**Table 1:** Metrics for Trajectory Evaluation.

Parameter	Primary role	Sensitivity characteristics	Suitable for detecting
RMSE	Overall error magnitude	High sensitivity to large deviations	Significant trajectory drift or tracking failure
SD	Stability measurement	Sensitive to variation across frames	Local instability and motion inconsistency
Mean	Bias detection	Low sensitivity to outliers, highlights long-term trends	Systematic bias in trajectory estimation

The experimental results confirm the effectiveness of our algorithm, with percentage improvement expressed as:

11 $$\begin{aligned} \theta =\frac{\lambda -\mu }{\lambda } \times 100\% \end{aligned}$$

Where $\theta$ represents the improvement of root-mean-square error, $\lambda$ represents the root-mean-square error value of the standard ORB-SLAM2, and $\mu$ is the root-mean-square error value of the improved algorithm.

#### A high-dynamics scene

**Table 2:** Comparison of absolute trajectory error (ATE) under high dynamics.

Sequence	ORB-SLAM2			Ours			Improvements (%)		
	RMSE	SD	Mean	RMSE	SD	Mean	RMSE	SD	Mean
fr3-w-h	0.4858	0.2684	0.4037	0.0256	0.0125	0.0227	94.74	95.34	94.37
fr3-w-r	0.3053	0.2246	0.2761	0.0057	0.0026	0.0048	98.14	98.85	98.27
fr3-w-s	0.8120	0.4165	0.6962	0.0145	0.0073	0.0129	98.21	98.25	98.15

**Table 3:** Translation comparison of relative pose error (RPE) under high dynamics.

Sequence	ORB-SLAM2			Ours			Improvements (%)		
	RMSE	SD	Mean	RMSE	SD	Mean	RMSE	SD	Mean
fr3-w-h	0.0381	0.0263	0.0273	0.0197	0.0113	0.0162	48.29	57.03	40.66
fr3-w-r	0.0371	0.0313	0.0196	0.0086	0.0054	0.0067	76.82	82.75	65.81
fr3-w-s	0.0544	0.0404	0.0362	0.0191	0.0112	0.0151	64.89	72.28	58.29

**Table 4:** Rotation comparison of relative pose error (RPE) under high dynamics.

Sequence	ORB-SLAM2			Ours			Improvements (%)		
	RMSE	SD	Mean	RMSE	SD	Mean	RMSE	SD	Mean
fr3-w-h	0.8650	0.5469	0.6703	0.5602	0.3133	0.4643	35.23	42.71	30.73
fr3-w-r	0.6593	0.5174	0.4086	0.2451	0.1317	0.2067	62.83	74.55	49.41
fr3-w-s	1.1048	0.7716	0.7909	0.5171	0.2726	0.4394	53.21	64.66	44.44

We conducted a comparative analysis using three datasets: freiburg3-walking-static (fr3-w-s), freiburg3-walking-halfsphere (fr3-w-h), and freiburg3-walking-rpy (fr3-w-r), with improvements expressed as percentage increases. The main evaluation metrics include Root Mean Square Error (RMSE), which measures the average deviation between predicted and true values, as well as Mean and Standard Deviation (SD), which assess the degree of deviation and data distribution, respectively. Table 2 shows that our method achieves over 90% improvement in all performance metrics of Absolute Trajectory Error. Tables 3 and 4 present relative trajectory errors in translation and rotation, demonstrating improvements across all aspects.

**Fig. 3:**
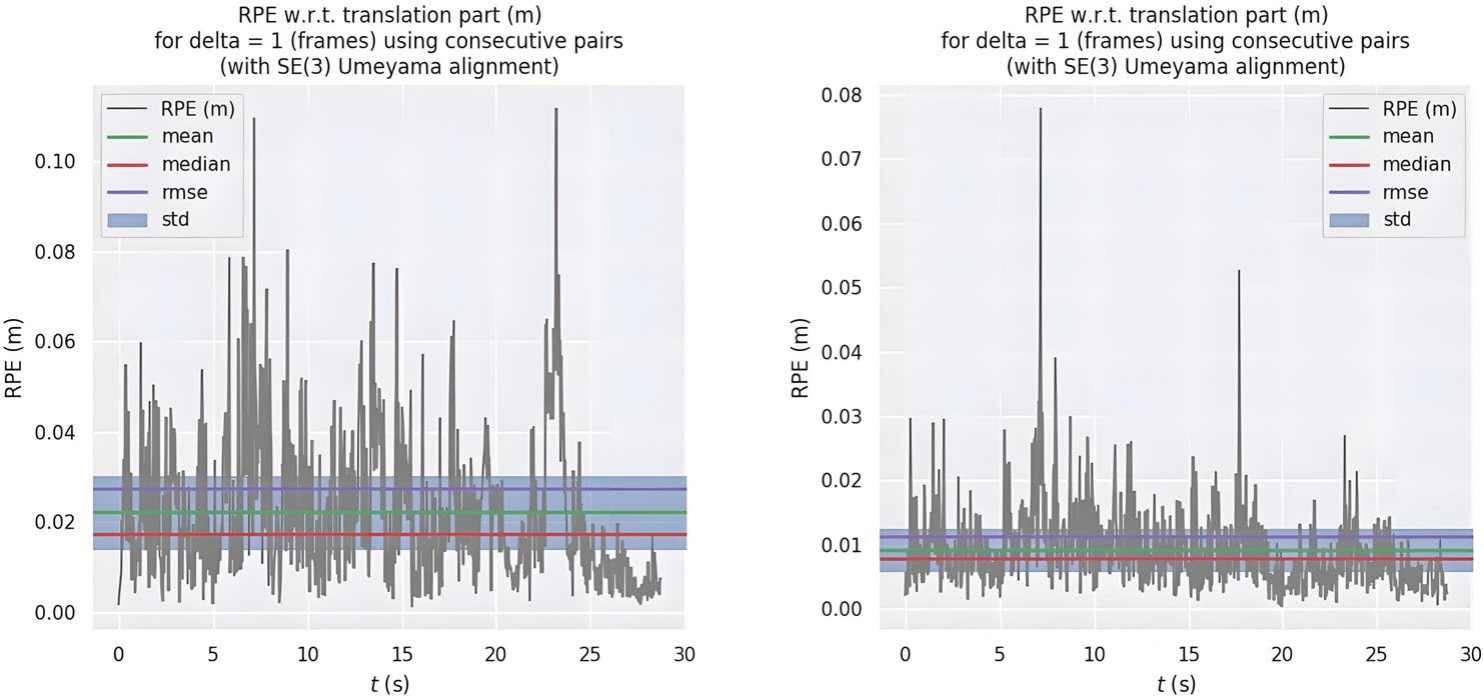
Fluctuations in relative path error in translation at high dynamics.

**Fig. 4:**
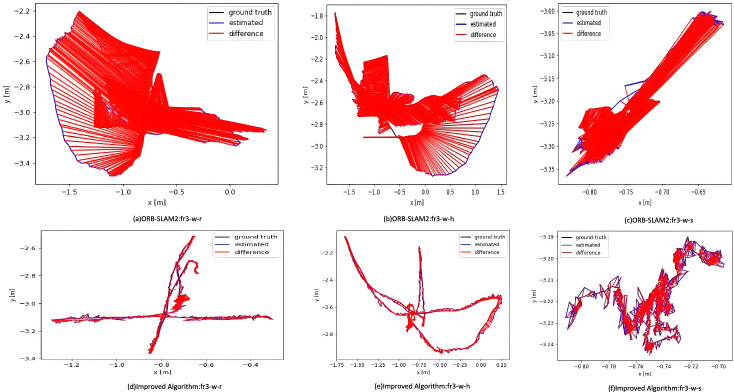
High dynamic trajectory comparison.

Figure 3 illustrates the relative translational path error fluctuations of ORB-SLAM2 (left) and the proposed algorithm (right). Our method shows more stable error fluctuations with a narrower range, keeping errors generally within 0.03 meters and a maximum below 0.08 meters. In contrast, ORB-SLAM2 exhibits greater fluctuation, with an average error around 0.04 meters and a maximum exceeding 0.1 meters. Figure 4 visualizes positioning accuracy, where black lines represent true paths, blue lines represent predicted paths, and red areas denote errors. Larger red areas indicate greater error. The comparison highlights that our improved algorithm demonstrates greater stability and robustness, particularly in high-dynamic sequences.

#### A low-dynamics scene

**Table 5:** Comparison of absolute trajectory error (ATE) under low dynamics.

Sequence	ORB-SLAM2			Ours			Improvements (%)		
	RMSE	SD	Mean	RMSE	SD	Mean	RMSE	SD	Mean
fr3-s-x	0.0134	0.0056	0.0121	0.0081	0.0043	0.0071	39.55	23.21	41.32
fr3-s-s	0.0097	0.0079	0.0086	0.0063	0.0027	0.0055	38.05	65.82	36.05
fr3-s-r	0.2935	0.2377	0.5442	0.0218	0.0135	0.0172	92.57	94.33	96.84

**Table 6:** Translation comparison of relative pose error (RPE) under low dynamics.

Sequence	ORB-SLAM2			Ours			Improvements (%)		
	RMSE	SD	Mean	RMSE	SD	Mean	RMSE	SD	Mean
fr3-s-x	0.0200	0.0118	0.0160	0.0143	0.0069	0.0124	28.51	41.52	22.51
fr3-s-s	0.0202	0.0021	0.0200	0.0084	0.0023	0.0123	58.42	-9.52	38.5
fr3-s-r	0.0332	0.0262	0.0227	0.0218	0.0178	0.0194	34.34	32.06	14.54

**Table 7:** Rotation comparison of relative pose error (RPE) under low dynamics.

Sequence	ORB-SLAM2			Ours			Improvements (%)		
	RMSE	SD	Mean	RMSE	SD	Mean	RMSE	SD	Mean
fr3-s-x	0.5891	0.3091	0.5016	0.4978	0.2589	0.4253	15.51	16.23	15.21
fr3-s-s	0.5019	0.1585	0.4763	0.3876	0.1563	0.3546	22.77	1.39	25.55
fr3-s-r	0.8499	0.5421	0.6545	0.7014	0.4488	0.5392	17.48	17.21	17.62

Referring to research on high-dynamic sequences, experiments were conducted on low-dynamic sequences (freiburg3-sitting-rpy (fr3-s-r), freiburg3-sitting-static (fr3-s-s), and freiburg3-sitting-xyz (fr3-s-x)). Tables 5, 6, and 7 present the results for absolute trajectory error, relative translational trajectory error, and relative rotational trajectory error, respectively. The results show that the improved algorithm achieves an improvement of up to 96.84% in absolute trajectory error for the fr3-s-r sequence, further demonstrating its high positioning accuracy in low-dynamic scenarios.

**Fig. 5:**
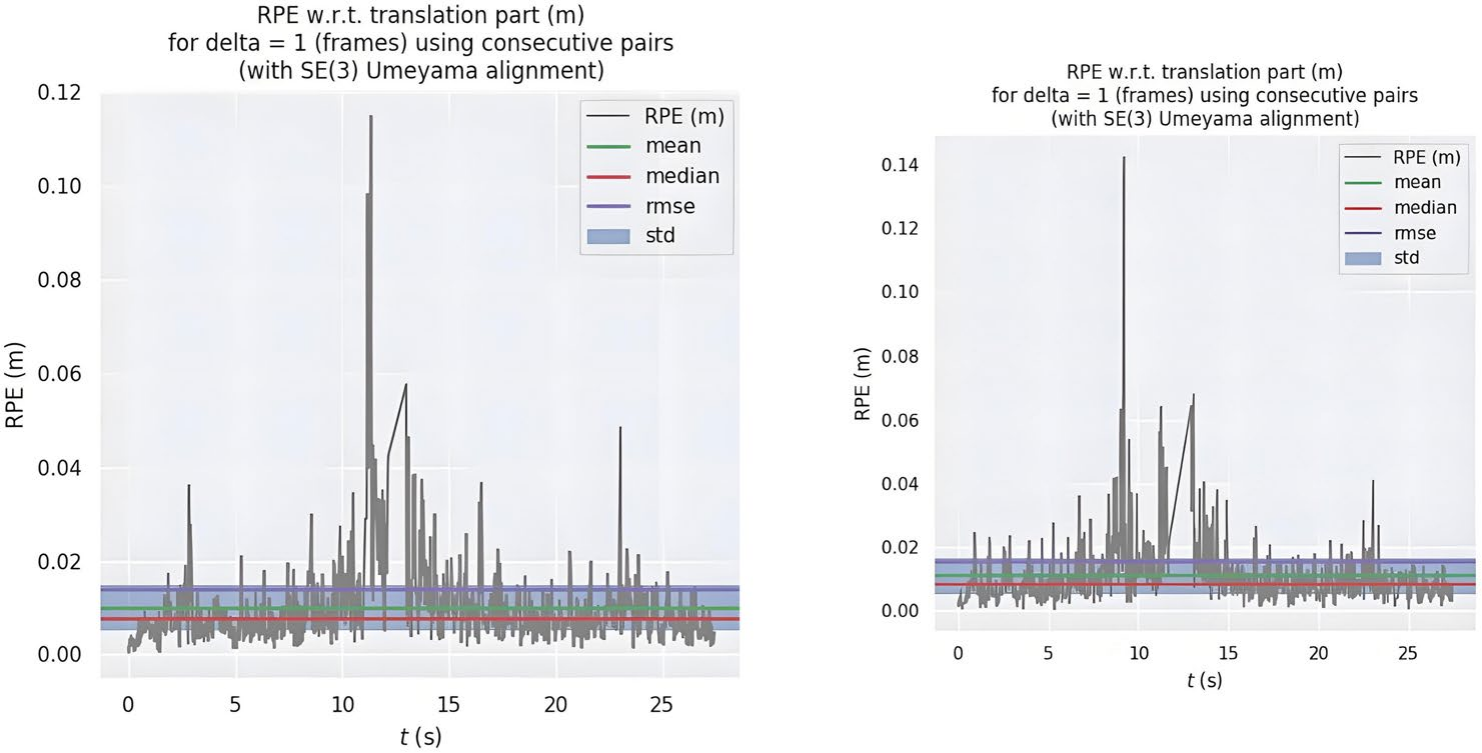
Relative path error fluctuations in translation at low dynamics.

**Fig. 6:**
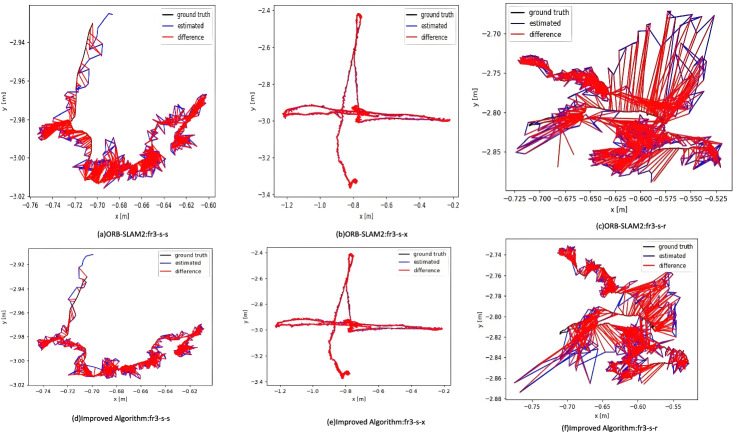
Low dynamic trajectory comparison.

Figure 5 shows the relative translational path error fluctuation of ORB-SLAM2 (left) and the proposed algorithm (right). Although the maximum error of the proposed algorithm is slightly higher than that of ORB-SLAM2, it maintains error fluctuations within 0.02 meters with a more uniform and stable distribution. The comparison in Fig. 6 indicates that the improved algorithm exhibits more balanced performance in dynamic scenarios, effectively addressing challenges from environmental changes, while ORB-SLAM2 shows limitations in adapting to such changes.

In summary, compared to ORB-SLAM2, the proposed algorithm demonstrates improved accuracy in both highly dynamic and low-dynamic scenarios, with particularly notable enhancements in highly dynamic environments. Key contributions affecting RPE and ATE include: 1) The enhanced quadtree-based feature extraction optimizes node splitting depth and prioritizes high-response features, ensuring uniform distribution while reducing computational redundancy in texture-rich regions, thereby significantly reducing localization errors caused by feature clustering. 2) The dynamic feature filtering mechanism, which combines semantic information and geometric constraints, effectively identifies and removes features on dynamic objects, directly minimizing their adverse impact on ATE. 3) The clustering-based matching strategy processes unmatched features between consecutive frames, expanding the search range while reducing mismatches caused by dynamic objects, thus stabilizing RPE. These synergistic improvements significantly enhance the system’s robustness and stability in dynamic scenes while maintaining real-time performance.

### Alation experiments

Ablation studies validated our algorithm by comparing the effects of removing deep learning, removing geometric constraints, and combining both. These setups comprehensively assess each module’s contribution, further confirming the effectiveness of our algorithm.

**Fig. 7:**
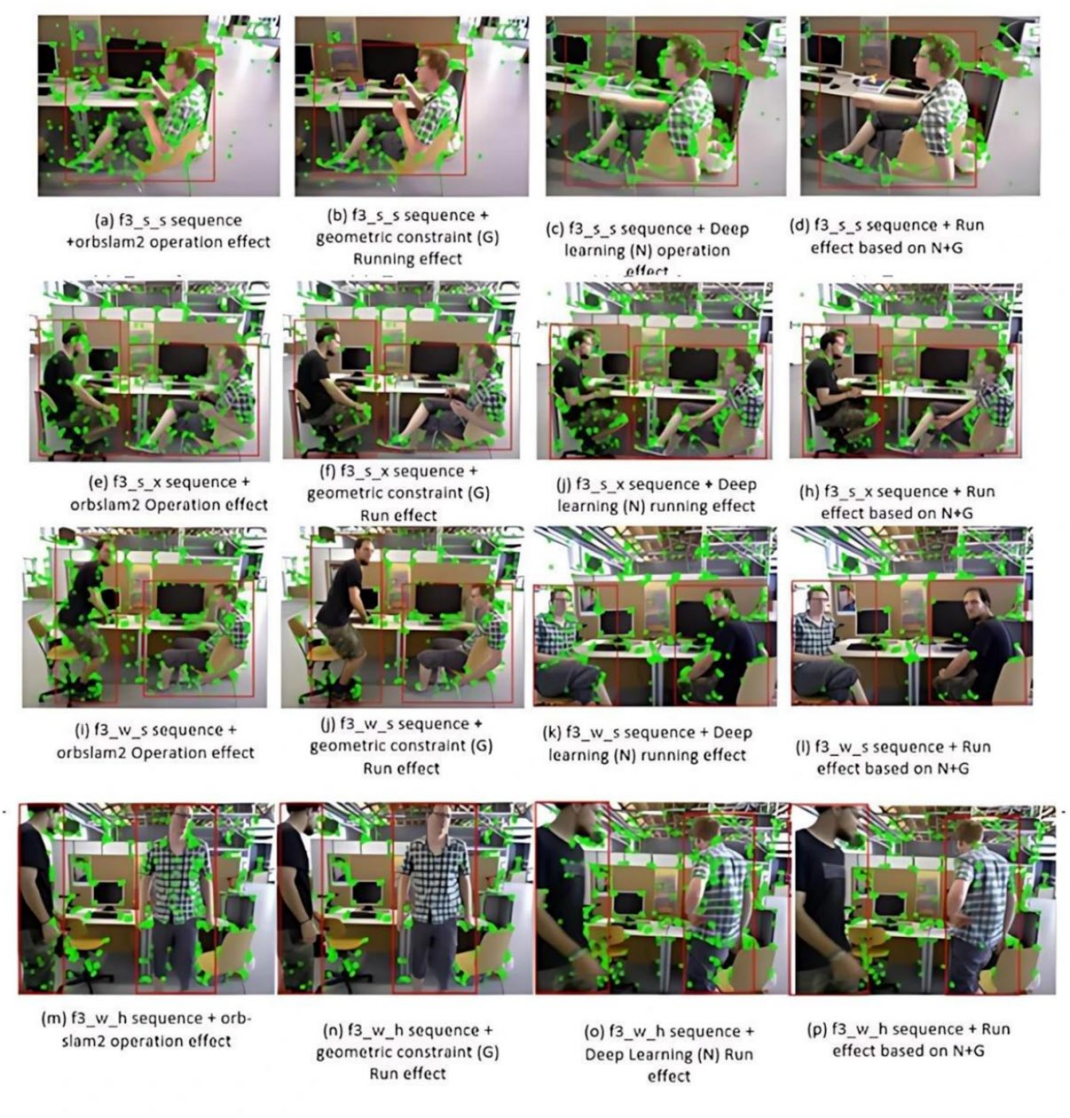
Comparison of image feature points extracted using different methods.

Figure 7 illustrates the ablation study results, analyzing the effects of removing deep learning, geometric constraints, and their combination. Subfigures (a), (e), (i), (m) show feature point extraction using the ORB-SLAM2 algorithm; (b), (f), (j), (n) depict results after removing geometric constraints; (c), (g), (k), (o) show results without deep learning; and (d), (h), (l), (p) display the combination of removing both. The first and second rows in Fig. 7 represent tests in low-dynamic environments, while the third and fourth rows correspond to tests in high-dynamic environments.

The results indicate that ORB-SLAM2 struggles with dynamic feature points in both low- and high-dynamic environments. In low-dynamic settings, geometric constraints provide limited improvement, while deep learning tends to misclassify static objects as dynamic, reducing localization accuracy. In high-dynamic environments, ORB-SLAM2 retains many dynamic points, with geometric constraints failing to identify distant dynamic objects and deep learning struggling with subtle movements. In contrast, the proposed algorithm, combining deep learning and geometric constraints, effectively detects and removes dynamic feature points, demonstrating superior robustness and accuracy across dynamic environments.

Table 8 compares the Root Mean Square Error (RMSE) in dynamic environments for the improved algorithm and two alternative approaches. The improved algorithm, combining deep learning and geometric constraints, achieves the lowest Root Mean Square Error (RMSE) in both low- and high-dynamic environments, outperforming methods relying solely on deep learning or geometric constraints, demonstrating superior accuracy and robustness in dynamic scenarios.

**Table 8:** Comparison of root mean square error (unit: m).

Sequence	Geometric constraint model	Deep learning model	Ours
fr3-w-s	0.0214	0.0212	0.0153
fr3-w-h	0.0359	0.0354	0.0254
fr3-s-s	0.0063	0.0076	0.0062
fr3-s-x	0.0086	0.0086	0.0084

## Conclusion and prospect

This paper proposes a feature detection and filtering algorithm tailored for dynamic environments to improve the localization accuracy and robustness of visual SLAM systems in complex environments. By improving the Qtree ORB feature extraction algorithm in the traditional ORB-SLAM2 system, the uniformity of feature point extraction is optimized. The method integrates semantic information and geometric constraints to effectively filter dynamic feature points. The approach prioritizes retaining high-response feature points, removes feature points from known dynamic objects, and addresses feature point matching issues using an expansion technique. Experimental results on the TUM dataset demonstrate that the proposed algorithm significantly enhances localization accuracy and system performance in dynamic environments.

However, despite the promising results in the current experiments, challenges remain in more complex dynamic environments. For instance, the system’s robustness may be impacted in scenarios involving multiple dynamic objects or complex interactions between dynamic objects and static backgrounds. In challenging outdoor scenarios characterized by intense illumination and densely distributed fast-moving objects, the proposed algorithm may experience trajectory drift. This is primarily caused by the degradation of feature detection and tracking reliability under strong lighting and rapid motion, which affects the accuracy of dynamic feature point removal and pose estimation. To address this limitation, future work will focus on integrating more illumination-invariant feature representations and employing more advanced dynamic object detection techniques to enhance system robustness.

In addition, incorporating multi-sensor fusion techniques, such as combining visual data with LiDAR and inertial measurement units, will be explored to mitigate the influence of harsh outdoor environments and improve overall trajectory stability. Furthermore, optimizing the real-time performance and computational efficiency of the algorithm, especially for resource-constrained devices, is an important area for future research. Finally, while the experiments in this paper were conducted on the TUM dataset, it is crucial to extend the method to larger-scale, more challenging real-world environments to ensure its broader applicability and stability across various dynamic scenes.

## Data Availability

The datasets used and/or analysed during the current study available from the corresponding author on reasonable request.
